# Mendelian randomization applied to Neurology: Promises and challenges

**DOI:** 10.1212/WNL.0000000000209128

**Published:** 2024-01-23

**Authors:** Eloi Gagnon, Iyas Daghlas, Loukas Zagkos, Muralidharan Sargurupremraj, Marios K. Georgakis, Christopher D. Anderson, Héléne T. Cronjé, Stephen Burgess, Benoit J. Arsenault, Dipender Gill

**Affiliations:** 1 Quebec Heart and Lung Institute, Laval University, Quebec, Canada; 2 Department of Neurology, University of California San Francisco, San Francisco, CA, USA; 3 Broad Institute of MIT and Harvard, Cambridge, Massachusetts; 4 Center for Genomic Medicine, Massachusetts General Hospital, Boston, Massachusetts, USA; 5 Department of Epidemiology and Biostatistics, School of Public Health, Imperial College London, London, United Kingdom; 6 Glenn Biggs Institute for Alzheimer’s & Neurodegenerative Diseases, University of Texas Health Sciences Center, San Antonio, TX; 7 Institute for Stroke and Dementia Research (ISD), University Hospital, LMU Munich, Munich, Germany; 8 Department of Neurology, Brigham and Women’s Hospital, Boston, MA, USA; 9 Department of Public Health, Section of Epidemiology, University of Copenhagen, Copenhagen, Denmark; 10 MRC Biostatistics Unit, University of Cambridge, Cambridge, UK; 11 Cardiovascular Epidemiology Unit, Department of Public Health and Primary Care, University of Cambridge, Cambridge, UK; 12 Department of Medicine, Faculty of Medicine, Université Laval, Québec (QC), Canada

## Abstract

The Mendelian randomization (MR) paradigm allows for causal inferences to be drawn using genetic data. In recent years, the expansion of well-powered publicly available genetic association data related to phenotypes like brain tissue gene expression, brain imaging and neurological diseases offers exciting opportunities for the application of MR in the field of neurology. In this review, we discuss the basic principles of MR, its myriad applications to research in neurology, and potential pitfalls of injudicious applications. Throughout, we provide examples where MR-informed findings have shed light on long-standing epidemiological controversies, provided insights into the pathophysiology of neurological conditions, prioritized drug targets, and informed drug repurposing opportunities. With the ever-expanding availability of genome-wide association data, we project MR to become a key driver of progress in the field of neurology. It is therefore paramount that academics and clinicians within the field are familiar with the approach.

## Introduction

Since the introduction of the term ‘Mendelian randomization’ in 2003^1^, the number of papers on theoretical and applied Mendelian randomization (MR) has increased exponentially. The growing popularity of this “gene-based hack that is revolutionizing epidemiology”^2^ is justified by three core attributes. First, the MR framework provides an opportunity to address causal questions using publicly available genetic summary data alone. Second, its application has successfully addressed multiple clinical inquiries across research areas, including the field of neurology. Third, MR provides a framework for prioritization of potential drug targets including for neurological indications. Despite considerable methodological advances in the last two decades, applied MR continues to be subject to common analytical and interpretive pitfalls. In this review, we present the MR framework, provide examples of its application in neurology, and discuss areas most vulnerable to bias and misinterpretation, where MR should be undertaken with caution.

## Data Sources

This narrative synthesis of evidence was based on literature searches of PubMed tailored by the authors’ expert knowledge and opinion. Episodically up to 24 July 2023, PubMed searches were conducted without date restriction using combination of “Mendelian randomization” and the following clinical related terms: “ischemic stroke”, “Parkinson’s,” “multiple sclerosis,” and “migraine.”, "Alzheimer’s disease", and "drug development".

## What is Mendelian randomization?

A traditional observational study faces two main challenges to assess causality: bias due to reverse causality and confounding. Reverse causality bias occurs when the observed association between an exposure and an outcome is due to a causal effect of the outcome on the exposure. Bias due to confounding on the other hand, occurs when an association is explained by a third factor that causes both the exposure and the outcome. These biases make it difficult to know for certain whether an association from an observational study represents a cause-and-effect relationship.

**Figure 1:**
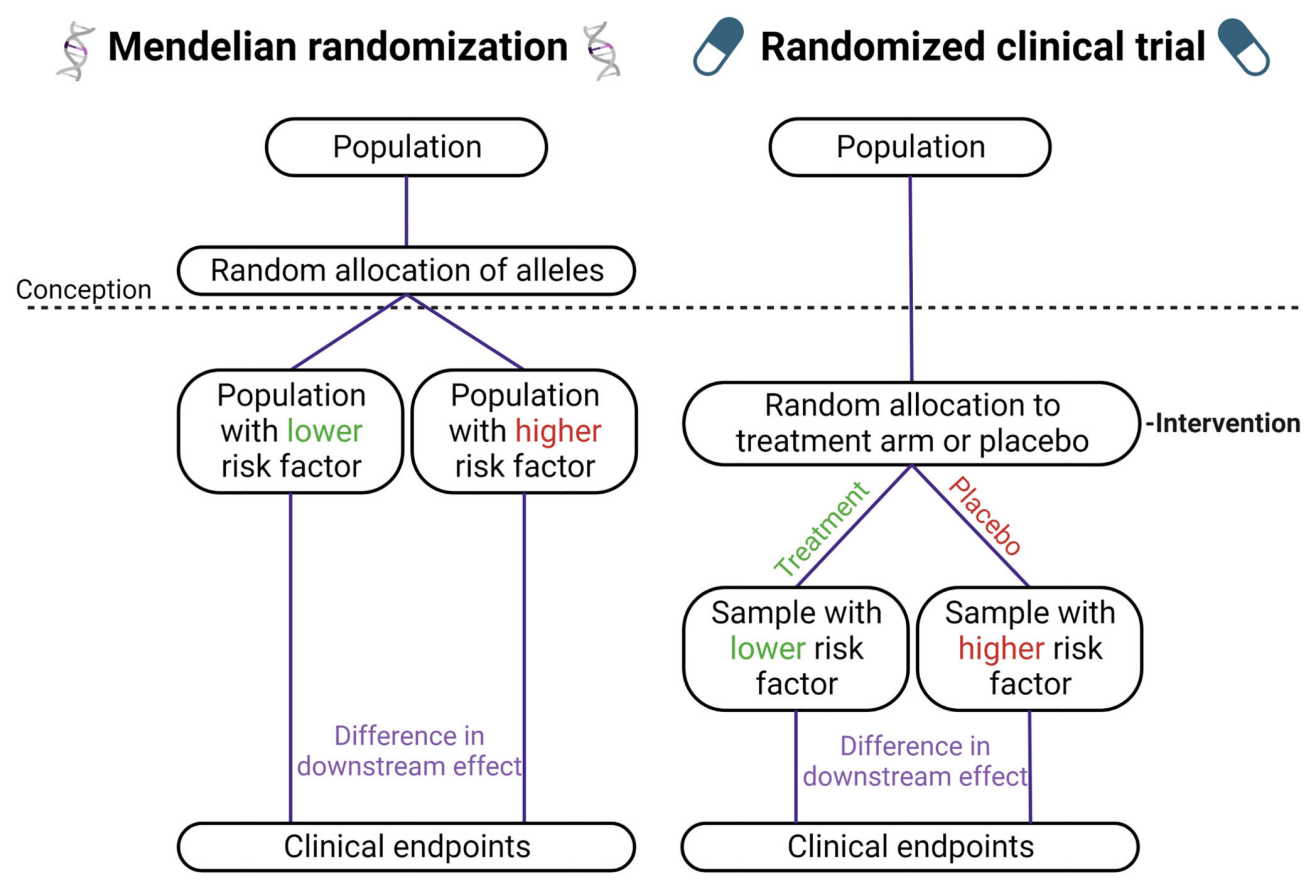
A parallel between randomized clinical trials and Mendelian randomization. The random allocation of alleles at conception offers a parallel to a “naturally randomized clinical trial”. Under certain assumptions, the random distribution of alleles in Mendelian randomization studies allows for the separation of individuals into groups that differ only with respect to the risk factor of interest. While the random allocation of alleles in Mendelian randomization studies happens at conception and leads to lifelong effects, the randomization in RCTs typically happens later in life and lasts a shorter time.

Mendelian randomization is an epidemiological method aimed at overcoming these limitations in order to draw causal inferences from observational data. To do this, MR relies on the logic of the instrumental variable, a statistical paradigm that has been developed for more than 50 years in areas outside genetics, aimed specifically at overcoming these biases^3^. In MR, genetic variants serve as instrumental variables to investigate whether a causal link exists between an exposure (e.g. low density lipoprotein) and an outcome (e.g. stroke). Since germline genetic variants are inherited at conception and are generally stable over time, they cannot be influenced by the development of a disease, thereby eliminating reverse causality bias. Furthermore, since germline genetic variants are inherited randomly at conception, they are largely independent of environmental and lifestyle confounding factors (Figure 1). For example, a patient’s body mass index does not influence the variant they carry at any genomic location.

**Figure 2:**
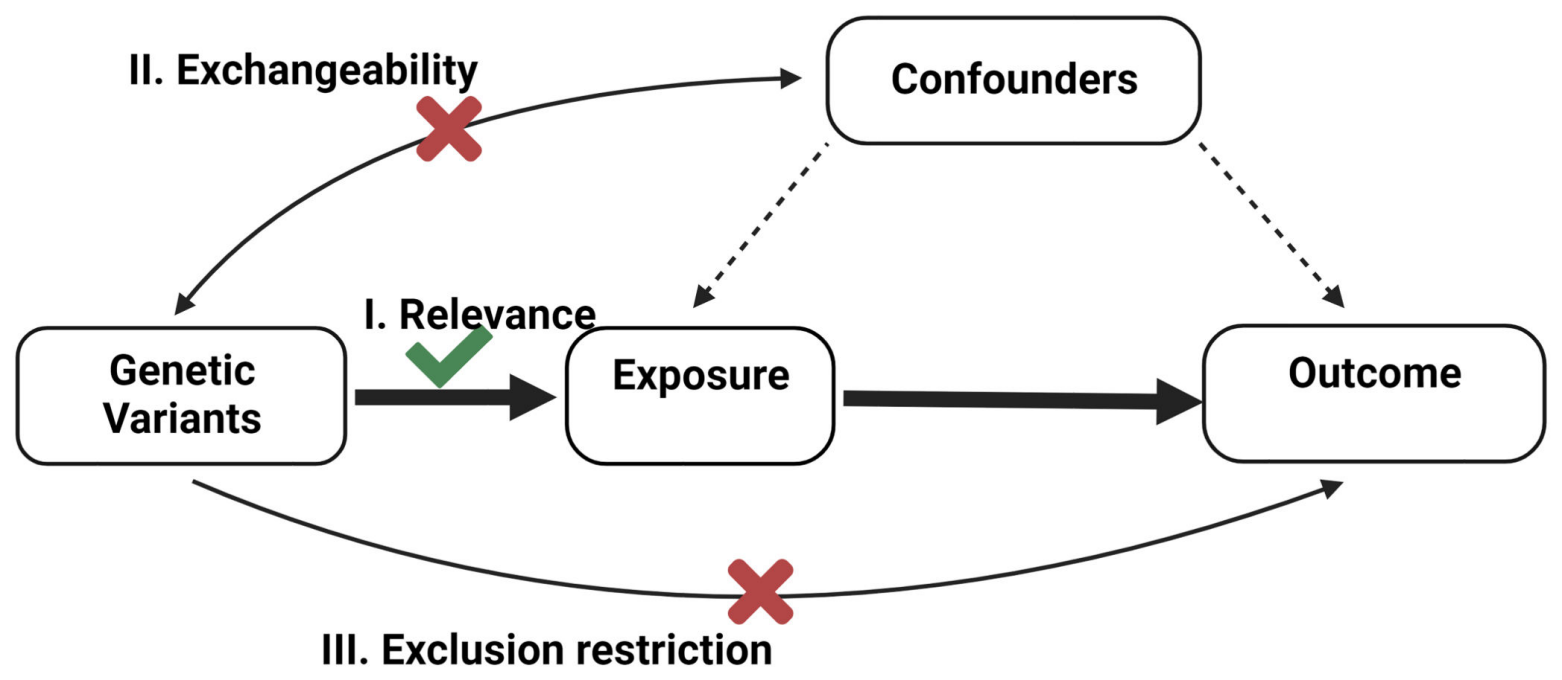
The three main assumptions of Mendelian randomization. First, the relevance assumption states that the genetic instrument must have a significant effect on the exposure variable. Second, the exchangeability assumption states that the genetic variant must not be associated with confounding factors. Third, the exclusion restriction assumption states that the genetic instrument must only affect the outcome through its effect on the exposure.

For MR to draw valid causal inference, the genetic instruments must meet three core assumptions. First, the genetic instrument must be strongly associated with the exposure variable (i.e., relevance). Second, the genetic variant must not be associated with confounding factors of the exposure-outcome association (i.e., exchangeability). Third, the genetic instrument must have no direct effect on the outcome, but instead only affect the outcome through the exposure (i.e., exclusion-restriction)^4^. If these three assumptions hold, then under further assumptions including those of linearity, the causal effect of the risk factor on the outcome can be calculated by dividing the association estimate of the genetic variant with the outcome by the association estimate of the genetic variant with the risk factor (Figure 2).

**Figure 3:**
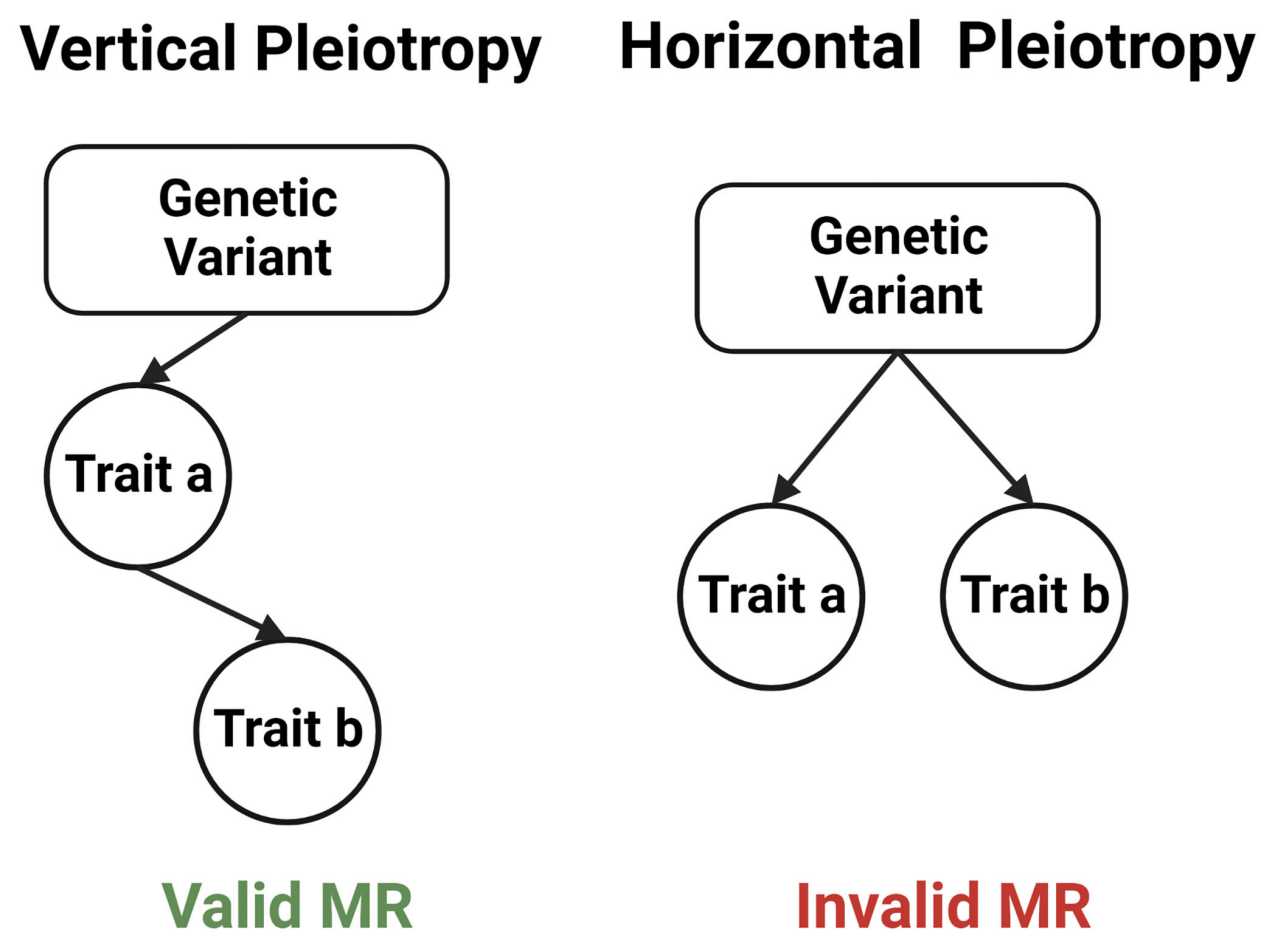
The distinction between horizontal and vertical pleiotropy. Vertical pleiotropy occurs when the genetic variant has a direct effect on the exposure, and an indirect effect on the outcome that is mediated by the exposure. Horizontal pleiotropy occurs when the genetic variant. Vertical pleiotropy does not invalidate Mendelian randomization results, but horizontal pleiotropy invalidates Mendelian randomization results as it transgresses the exchangeability assumption.

In practice, only the first assumption is verifiable. Assumption two is thought to generally be adequately satisfied by controlling for genetic ancestry but may still be violated by uncontrolled population stratification or by ‘assortative mating,’ whereby the traits or genetic variants under study influence partner selection. The third assumption is the strongest assumption and the most likely to be invalidated by pleiotropy. Pleiotropy in this context means that a genetic variant influences multiple traits (Figure 3). Vertical pleiotropy describes a scenario where a genetic variant has a direct effect on the exposure and an indirect effect on the outcome that is mediated by the exposure. This does not induce bias and the IV assumptions for the MR analysis hold. In contrast, horizontal pleiotropy describes a scenario where a genetic variant exerts direct effects on both the exposure and other parallel pathways that may influence the outcome. This does induce bias and threatens validity of causal inference from an MR analysis. Cautious instrument selection and appropriate MR study design is paramount to minimizing the influence of this bias.

## Instrument selection and sensitivity analyses (statistical versus biological approaches)

The most critical decision in an MR investigation is the choice of which genetic variants to use as IVs. Broadly, there are two strategies to select genetic instruments: a biologically driven approach and a statistically driven, biologically-agnostic approach ^5^. In a biologically driven approach, variants are selected from genomic regions with a known biological link to the exposure. This method often uses fewer genetic instruments (sometimes just one), which are less likely to be pleiotropic. For example, a *cis*-MR approach leverages genetic variants located in the genetic region encoding for the protein or gene transcript of interest. These genetic variants may influence proteins levels more directly and are therefore more likely to be valid IVs. The disadvantage of this method is that it requires pre-existing biological knowledge, often offers fewer IVs than a genome-wide approach, and precludes the use of certain sensitivity analyses that require multiple genetic variants.

By contrast, a statistically driven approach typically includes all the variants robustly associated with the exposure (often employing a p-value below the genome-wide significance threshold of 5 x 10^-8^) across the entire genome, even if their biological function is unknown. Eligible genetic variants are subsequently pruned to remove correlated variants (i.e. variants that are inherited together). The statistically driven approach decreases reliance on a single genomic region and includes more variants, thus potentially increasing statistical power. However, it also increases the odds of including invalid, horizontally pleiotropic instruments, which might introduce bias into the analysis.

Statistical sensitivity analyses can be used to compensate for the inclusion of invalid instruments by performing robust MR methods that require less strict model assumptions to make valid causal inference. For example, the weighted median method can provide a valid causal estimate even if up to half of the information for the analysis comes from genetic variants comprising the IV violate one or more requisite assumption^6^. Generally, it is recommended to perform a variety of robust MR methods that operate in statistically distinct ways and rely on different assumptions about the underlying nature of pleiotropy. Directionally consistent causal estimates across different robust MR methods provide confidence about the validity of the observed causal link. A detailed overview of the frequently used robust MR methods and their core assumptions is available^7^, and can be used to assist in the interpretation of MR results.

Because biologically driven approaches often use few genetic variants, they are not always amenable to statistical sensitivity analyses that require multiple uncorrelated variants. When a single genetic variant is used, it is recommended to perform colocalization as a sensitivity analysis, such as is implemented in the method *Coloc*^8^. Coloc is a Bayesian algorithm that evaluates the posterior probability of two traits sharing a single causal variant^9^. Coloc is particularly useful to evaluate a specific form of confounding where the causal genetic variant for the exposure and the causal genetic variant for the outcome are different, but inherited together. A strong colocalization posterior probability of a shared causal variant provides additional evidence for a causal association by indicating that confounding by LD is unlikely and the MR inference is unbiased.

The research question informs whether it is better to use more genetic variants and more complex methods (i.e., statistically driven approach), or a more curated set of variants and simpler methods (i.e., biologically driven approach). When investigating proximal gene products such as proteins or gene expression levels as the exposure, a biologically driven approach will likely yield a more valid inference. When investigating polygenic traits such as body mass index, statistically driven approaches may be more appropriate, particularly as such traits are heterogeneous with multiple distinct underlying pathways. Both instrument selection methods have advantages and disadvantages, and their combined use can help triangulate the true causal estimate ^10^.

## Mendelian randomization study of drug targets

Bringing a new drug to the market can cost, on average, anywhere from hundreds of millions to over a billion dollars^11^. The cost of drug development is exacerbated by the high failure rate (up to 90% of drug candidates never make it to market, even after a successful phase 1 clinical trials)^11^. The most common reason for failure is a lack of drug efficacy, which accounts for 40–50% of terminated clinical trials. This means that even after promising results from preclinical studies and early-phase clinical trials, the drug does not demonstrate the intended therapeutic effect in humans and is therefore discontinued, highlighting the poor translatability of findings from animal models to humans^12,13^.

The MR paradigm is a promising strategy to help accelerate and reduce the cost of the drug development pipeline. Indeed, MR studies are relatively inexpensive to conduct—particularly compared to a large, randomized controlled trials (RCTs)—and can provide evidence that can be used to prioritize targets worth investigating in RCTs. Drug target MR relies on the previously described *cis*-MR method, where genetic IVs are obtained from the genomic region of the target of interest.

For efficacy, MR can test whether a particular target is causally linked to a specific disease or condition. For safety, the MR framework can be implemented to test the effect of the target on thousands of clinical traits from electronic health care repositories. Second, drug target MR can inform on drug repurposing opportunities by leveraging the genetic predictors of targets of medications with published safety profiles to identify novel indications for the drug^14^. Finally, with individual-level data, it is possible to test for the interactions between two drug targets using MR methods, as in a 2x2 factorial RCT^15^.

## Applications of Mendelian randomization to Neurology

The MR paradigm has been used to investigate clinical hypotheses across all subspecialties of neurology. The guidance offered by MR analyses regarding disease pathophysiology, causal effects, and potential drug targets, are particularly important in neurology given the long, slow progression of many neurological diseases (e.g., Alzheimer’s disease and related dementias) which poses unique challenges to clinical trials. As in all specialties, time and money are limited and tools to predict the success of clinical interventions and pharmacologic therapies are necessary. Here, we highlight examples of applications of MR across several subspecialties of neurology.

### Ischemic stroke

MR has revolutionized the exploration of risk factors for different ischemic stroke subtypes. Large trials typically do not have the scope to phenotype stroke cases down to their etiological subtypes. However, because of the recent availability of GWAS data for stroke subtypes^16^, it is now possible to dissect their risk factor profiles with MR. For example, MR analyses shown that elevated low-density lipoprotein cholesterol selectively increases the risk of large-artery atherosclerotic stroke, but not the risk of cardioembolic or small vessel stroke^17^. This finding addressed a clinical knowledge gap, as the largest clinical trial of statins in stroke was conducted without stroke subtyping and excluded cases of cardioembolic stroke^18^.

With respect to drug target prioritization, the benefits of lowering blood pressure^19^ and cholesterol^17^ for stroke prevention were retrospectively identified by MR studies after large RCT were conducted. For example, one MR study compared the effects of genetically proxied calcium channel blockade versus beta blockade on the risk of stroke^19^. This investigation found that genetically proxied blood pressure reduction through calcium channel blockade significantly lowered the risk of ischemic stroke, while genetically proxied beta blockade had no effect on stroke risk. These observations align with the current clinical guidelines for the treatment of hypertension that advise against use of beta-blockers as first-line treatments for hypertension due to their inferior efficacy for reducing stroke risk^20^.

Several MR studies provided evidence for a causal effect of IL-6 signaling on ischemic stroke, particularly large artery, and small vessel stroke ^19,21^, supporting the candidacy of IL-6 signaling as a target for vascular prevention. Trials targeting IL-6 signaling are under way so definitive evidence are yet to come. Recent MR data indicates that 67% of the effects of genetically downregulated IL-6 signaling on large artery atherosclerotic stroke could be mediated by decrease in CXCL10 circulating levels^22^. These results suggest CXCL10 to be a potentially causal mediator of atherosclerosis and as such might serve as a promising, potentially more specific drug target than IL-6 signalling.

Finally, MR has provided support for a novel drug target to be used for stroke prevention: the coagulation factor eleven (FXI). Higher genetically predicted circulating levels of FXI were shown in an MR analysis to increase ischemic stroke risk^23^. Subsequent studies recapitulated this finding and demonstrated that these effects were specific to cardioembolic stroke ^24,25.8,9^. Excitingly, genetically predicted FXI inhibition does not appear to increase the risk of intracranial or extracranial bleeding, further supporting the hypothesis that FXI inhibition may be a safer therapeutic strategy compared to established anticoagulants such as coagulation factor Xa inhibitors^24^. MR has also been used to investigate the outcomes of intracerebral hemorrhage and subarachnoid hemorrhage and results from these studies are partially summarized elsewhere ^26^.

### Alzheimer’s disease

Investigating the pathophysiological basis of neurological conditions, such as Alzheimer’s disease, ideally requires access to brain tissue, but this has historically not been feasible at a large scale. New GWAS of brain protein levels now permits detailed evaluation of the brain pathophysiology of neurological conditions. For example, Robins et al., preformed a GWAS of 8,356 proteins measured by liquid chromatography coupled to mass spectrometry in the dorsolateral prefrontal cortex of 330 postmortem brain donors^27^. Using this dataset, they perform a brain proteome-wide MR and identified 13 genes whose cis-regulated brain protein abundance was associated with Alzheimer’s disease^28^. Similarly, Yand et al. performed a GWAS of 1,305 protein levels in the cerebrospinal fluid (*n* = 971) and parietal cortex (n=458) using an aptamer-based platform. Using Mendelian randomization, the authors identified three proteins implicated in Alzheimer’s disease risk in the cerebrospinal fluid and seven in the parietal lobe.

### Parkinson’s disease

MR analyses have been particularly useful in distinguishing causal from non-causal risk factors for Parkinson's disease (PD). Observational studies have consistently shown that serum levels of vitamin D and urate are inversely associated with the risk of PD^29,30^. However, MR analyses have not supported a causal effect of either of these biomarkers on PD risk, suggesting that these observational associations may be confounded or attributable to reverse causation ^30,31^. MR analyses have also been used to identify novel risk factors of PD, such as plasma levels of alpha-L-iduronidase (IDUA). Leveraging data from a hypothesis-free scan of the plasma, cerebrospinal fluid, and brain proteome, IDUA was identified alongside 34 other proteins as having a potentially causal association with PD^32^. Alpha-L-iduronidase is responsible for the degradation of glycosaminoglycans in the lysosome, an organelle which is implicated in at least monogenic forms of PD^32^.

### Multiple sclerosis

The MR framework has been used to weigh in on longstanding epidemiological controversies related to multiple sclerosis (MS). One example includes the replicated MR evidence that higher levels of vitamin D levels causally lower the risk of MS^33^. Additionally, MR analyses have shown that the effect of adiposity on MS risk varies across the life course. Specifically, MR findings indicated that genetically proxied adiposity in childhood, but not adulthood, is causally associated with an increased risk of MS^33^. In contrast, MR analyses have not supported an effect of these risk factors on MS severity^34^.

With respect to drug repurposing opportunities, one MR analysis identified interleukin-6 signaling as a potential causal risk factor for MS^35^. Subsequent mediation analysis revealed that altered interleukin-6 signaling explained more than 40% of the observed effect of body mass index on MS risk^35^. This example demonstrates how MR may simultaneously provide mechanistic insights for disease and identify novel causal risk factors and therapeutic targets.

### Migraine

Migraine is a common, disabling neurological disorder that remains poorly understood. In an exploration of the causal relevance of prior epidemiological findings linking poor sleep to migraine ^36^, MR analyses have identified both insomnia and difficulty awakening as potentially causal risk factors ^37^. This substantiates the prioritization of treating sleep disturbances when treating migraine. Another therapeutically relevant advance in migraine research is the potentially causal relationship between multiple coagulation factors and migraine risk, identified using MR analyses^38^. This work showcased the dual role that MR can play by indirectly providing evidence that microemboli may play a role in the etiology of migraine and by prioritizing drug targets.

These examples highlight the myriad applications of MR across subspecialties of neurology. Many neurological diseases have yet to be investigated in MR analyses due to the paucity of large genome-wide association studies for certain phenotypes. This limitation precludes the detailed investigation of many disease subtypes (e.g., migraine with aura), disease severity (e.g., migraine frequency) and disease progression or recovery (e.g., motor recovery in stroke). The increasing availability of genetic data obtained from nervous system tissue, such as cerebrospinal fluid and neuronal cells, also present exciting opportunities for the application of MR to neurology. These large-scale data will allow more precise mechanistic insights and predictions regarding the efficacy of brain drug targets. Finally, the increasing availability of sex-stratified genetic data will facilitate a clearer understanding of how risk factors and mechanisms of disease may differ by sex.

## Common pitfalls of applied MR analyses

In this section, we discuss some common pitfalls in applied MR analyses. This is by no means an exhaustive list of all the potential challenges in applied MR, but rather a highlight of some frequently encountered issues that we encourage practitioners to consider carefully.

### Exposure and outcome specification

It is essential that the exposures being instrumented, and the outcomes of interest are defined fully and accurately. Exposure definition is particularly important in MR studies of binary traits (e.g. case/control)^39^. In these cases, MR can estimate the effect of disease liability (e.g. risk of stroke) an the outcome of interest. This is distinct from conventional epidemiologic studies where one may directly estimate the association of disease status with an outcome. MR studies considering binary or dichotomized exposure should be careful to communicate results as the association of genetic liability to the exposure with the considered outcomes, rather than the effect of the exposure.

### Instrument selection

Genetic variants employed as IVs should reflect variation in the exposure being studied. This issue created controversy in an MR study that aimed to investigate the effects of the anti-diabetic drug metformin by selecting genetic instruments through their association with circulating levels of growth differentiation factor 15^40^. As several letters in response to this manuscript pointed out^41^, this MR study was in fact investigating the causal effect of circulating growth differentiation factor 15 levels, rather than metformin use per se, and the two are not equivalent nor interchangeable.

Minimizing the biasing effect of pleiotropy is an important consideration when selecting instruments for MR analyses. For exposures that are influenced by environmental and lifestyle factors, confounding by pleiotropic pathways are inherent to instrument selection and should be addressed appropriately. For example, in an MR study aimed at investigating educational attainment, there is likely to be pleiotropy through associations of the genetic IVs with intelligence^42^. Where such pleiotropic pathways are known, and relevant genetic association data are available, multivariable MR – an MR technique evaluating more than one exposure – may be used to adjust for potential bias^43^.

Finally, biological insight may be used to inform instrument selection. For example, when studying the effect of caffeine, it is more appropriate to instrument these effects using genetic variants that predict plasma caffeine levels (i.e., the physiological presence of the bioactive component of interest) than variants affecting caffeine consumption (i.e., the behavioral trait). The latter is likely less biologically relevant, and is more vulnerable to biasing pleiotropy through relation with pleiotropic pathways such as dietary preferences, socio-economic status and lifestyle^44^. For plasma caffeine levels specifically, instrument validity can be further optimized by selecting genetic variants that are located in genes related to caffeine metabolism instead of a genome-wide approach.

### Shared etiology versus causal effects

Similarly, biological understanding is required to distinguish shared etiology from causal effects. For example, if the genetic variants employed as IVs for an exposure relate to an upstream trait that causes both the exposure and the outcome under consideration, then any apparent MR association may be attributable to a shared etiology for the exposure and the outcome, rather than a causal effect of the exposure on the outcome. This phenomenon may be at play for MR analyses investigating the causal effect of perivascular space burden on the risk of cerebral small vessel disease^45^, or of white matter hyperintensity volume on the risk of stroke or Alzheimer’s disease^46^.In these cases, common risk factors may be underlying the exposures and the outcomes. If a common shared etiological factor is suspected, its confounding effect can be tested using multivariable MR.

### Studies of disease progression

MR studies investigating the effects of an exposure on the risk of disease progression (or recovery) are vulnerable to collider bias if the exposure also affects disease risk^47^. Collider bias is a subtle form of bias that may emerge when analyses are performed in a ‘case-only’ dataset. Following the release of the genome-wide association summary data for functional recovery after stroke^48^, several MR studies have attempted to unravel potential interventions that favorably impact stroke recovery^49^. Results from these investigations should be interpreted cautiously, as the described collider bias can produce false positive findings. Although there are methods tailored to detection and correction for collider bias^47^, their practical application is not always feasible, and they remain sparsely implemented.

### Considerations for drug target MR

There are various pitfalls that are specific to MR analyses aimed at investigating drug targets perturbation. For example, some drug targets have multiple modes of action. This point is well illustrated by glucagonlike peptide 1 receptor (GLP1R), which is likely exerting effects on bodyweight and glycemic control through distinct mechanisms ^8^. As such, selecting genetic variants in the *GLP1R* region based on their statistical association with type 2 diabetes mellitus liability may only allow for the investigation of effects mediated through mechanisms of glycemic control, and exclude those related to other GLP1R effects, such as lowering body weight^50^.

A particular note of caution for drug target MR is that this approach cannot be used to study compound-specific pharmacological effects, but only the effect of perturbing the drug target. To illustrate this point, MR analyses have supported the effects of perturbing cholesteryl ester transfer protein on reducing risk of cardiovascular disease^51^. However, whether this finding translates to clinical trials will be affected by their study design, including the target population and the duration and intensity of treatment, as well as the pharmacological compound being studied and its magnitude of effect on the target ^63,64^.

Finally, as with all MR analyses, it is imperative to communicate that the analytic paradigm reflects the cumulative effect of small lifelong genetically predicted differences in the exposure. This is in contrast to clinical interventions which take place over a shorter time period and may result in much larger changes in the level of the exposure of interest (e.g. complete inhibition of a protein). Thus, results from MR analyses will not precisely predict the size of the effect of an intervention in clinical practice–these are typically of larger magnitude and over a much shorter period^54^.

### Future Perspectives

New opportunities for deeper understanding of neurological disease mechanisms come from the falling cost and increasing scalability of proteomics technologies, transcriptomics technologies, and imaging modalities. GWAS on brain protein levels ^32,66^, cerebrospinal protein levels^55^, brain gene expression^56^, brain magnetic resonance imaging^57^ and brain single-cell gene expression^58^ are, or will soon be, publicly available. These datasets can be leveraged to inform on pathophysiology of neurological condition.

The increasing power of GWAS will breach new frontiers in the study of rarer neurological conditions where etiological risk factors are not as well understood as for more prevalent conditions. GWAS have already uncovered the first loci for less common neurological conditions such as amyotrophic lateral sclerosis, cerebral palsy, dystonia, autoimmune encephalitis, Guillain-Barre syndrome, and hereditary ataxia. The application of Mendelian randomization to these disease outcomes may result in the identification of novel, modifiable risk factors.

Finally, Mendelian randomization methods are rapidly evolving. For example, the application of Mendelian randomization to disease progression while correcting for collider bias is an area of intense methodological development^60^. Similarly, methodologies to mitigate bias due to horizontal pleiotropy are continuously being developed and will result in more reliable causal inference.

## Conclusion

The integration of MR into neurology-oriented research has offered a solid foundation for identifying causal risk factors, advancing our understanding of the pathophysiology of neurological diseases, and assisting in the prioritization of drug targets. To reap its full benefit, researchers must conduct MR investigation carefully and include biological insights through all stages of analysis. As the field of MR in neurology continues to evolve, it is our hope that academics and clinicians will fully embrace its potential and use it constructively in efforts to serve patients.

## Glossary

FXI: coagulation factor eleven
GLP1R: glucagonlike peptide 1 receptor
IDUA: alpha-L-iduronidase
IVW: inverse-variance weighted
LD: linkage disequilibrium
MR: Mendelian randomization
MS: multiple sclerosis
PD: Parkinson’s disease
